# The Anti-Lung Cancer Activities of Steroidal Saponins of *P. polyphylla* Smith var. *chinensis* (Franch.) Hara through Enhanced Immunostimulation in Experimental Lewis Tumor-Bearing C57BL/6 Mice and Induction of Apoptosis in the A549 Cell Line

**DOI:** 10.3390/molecules181012916

**Published:** 2013-10-17

**Authors:** Yu Li, Jun-Fei Gu, Xi Zou, Jian Wu, Ming-Hua Zhang, Jun Jiang, Dong Qin, Jin-Yong Zhou, Bao-Xin-Zi Liu, Yun-Tao Zhu, Xiao-Bin Jia, Liang Feng, Rui-Ping Wang

**Affiliations:** 1 Department of Oncology, The Affiliated Hospital of Nanjing University of Chinese Medicine, No.155, Hanzhong Road, Nanjing 210029, China; E-Mails: liyu.emilee@gmail.com (Y.L.); zxvery@126.com (X.Z.); czcyg@sina.com (J.W.); jinyongzhou@126.com (J.-Y.Z.); liubaoxinzi@hotmail.com (B.-X.-Z.L.); 2 Key Laboratory of New Drug Delivery Systems of Chinese Meteria Medica, Jiangsu Provincial Academy of Chinese Medicine, Shi Zi street 100#, Hongshan road, Jiangsu, Nanjing 210028, China; E-Mails: gujunfei0123@126.com (J.-F.G.); jdyx0701.111@163.com (M.-H.Z.); xuyan9323@126.com (J.J.); search_winter@sohu.com (D.Q.); jxiaobin2005@hotmail.com (X.-B.J.); 3 Department of Oncology, The Frist Affiliated Hospital of Guangxi University of Chinese Medicine, Guangxi, Nanning 530001, China; 4 No. 1 Clinical Medical College, Nanjing University of Chinese Medicine, Nanjing 210029, China; E-Mail: zyt8548@126.com

**Keywords:** *P. polyphylla* Smith var. *chinensis* (Franch.) Hara, steroidal saponins, immunostimulation, inflammation, apoptosis

## Abstract

*P. polyphylla* Smith var. chinensis (Franch.) Hara (PPSCFH) has been used as medicinal Paris for the prevention and treatment of cancers in China for thousands of years. Its main components, steroidal saponins (PRS), have been confirmed to inhibit tumor growth. In the present study, the immunostimulation of PRS was investigated in Lewis bearing-C57BL/6 mice while the induction of apoptosis in A549 cells was also studied. The treatment with PRS (2.5, 5.0 and 7.5 mg/kg) significantly inhibited tumor, volume, and weight in the C57BL/6 mice. The rates of inhibition of PRS (at 2.5, 5.0 and 7.5 mg/kg) were 26.49 ± 17.30%, 40.32 ± 18.91% and 54.94 ± 16.48%, respectively. The spleen and thymus indexes were increased remarkably, while the levels of inflammatory cytokines including TNF-α, IL-8 and IL-10 in serum were decreased according to ELISA assays. For A549 cells, Hoechst 33342 staining and annexin V/PI by flow cytometry showed that PRS (0.25, 0.50 and 0.75 mg/mL) induced nuclear changes of A549 cells with DNA condensation and fragmentations of chromatin, as well as inducing apoptosis. Furthermore, PRS could also attenuate the over-generation of intracellular ROS. Western blotting analysis showed a significant decrease on the expressions of proinflammatory cytokines MCP-1, IL-6 and TGF-β1, as well as cell adhesion molecule ICAM-1, by treatment with PRS. Our results demonstrated that the inhibition of PRS on tumor growth might be associated with the amelioration of inflammation responses, induction of apoptosis, as well as the decrease of ROS. These results suggested that PRS implied a potential therapeutic effect in the lung cancer treatment.

## 1. Introduction

Lung cancer has been regarded as a leading cause of cancer-related mortality throughout the World. Its occurrence and development are associated with a variety of factors, including oxidative stress, apoptosis, immune factors disorders, dysfunction of lung epithelial cells, inflammation, *etc*. There is mounting evidence that the immunostimulation response triggered by the toxicity of endogenous and exogenous factors plays an important role in the pathogenesis of lung cancer [1]. Recently, pro-inflammatory cytokines, such as interleukin (IL)-10 and IL-8, have been proposed as mediators of the development and progression of lung cancer [2]. The high concentrations of cytokines showed the presence of a neutrophilic inflammation in non-small cell lung cancer (NSCLC) patients [3]. Recent studies showed that the multiple cytokines produced by tumor cells contributed to various effects on antitumor immune responses and tumor growth [4].

The benefical effects of traditional Chinese medicine on the prevention and treatment of tumors have attracted much attention. *P. polyphylla* Smith var. *chinensis* (Franch.) Hara (PPSCFH), a medicinal herb, has been used traditionally in China for many years for the prevention and treatment of tumors due to its anti-tumor activity. Phytochemical study showed that its main components, steroidal saponins (PRS), displayed a potential cytotoxicity against various tumor cells, such as CCRF leukemia cells, ECA109 esophageal cancer cells, CaEs-17 cells, human promyelocytic leukemia HL-60 cells, human liver carcinoma HepG-2 cells, human gastric cancer BGC-823 cells, human colon adenocarcinoma LoVo cells and SW-116 cells [5,6,7,8]. Recently, it has also been found that PRS can induce tumor cell apoptosis and inhibit the migration in murine lung adenocarcinoma *in vitro* and *in vivo* [9]. Many studies have suggested that the active compounds of PRS, such as polyphyllin I and polyphyllin D, exhibited antitumor ability in NSCLC cells [10,11,12]. However, the immunomodulatory and inducing apoptosis activities of PRS on lung cancer remains unclear. Therefore, the aim of the present study was to evaluate the lung cancer-related immunomodulatory and apoptosis inducing effects of PRS in tumor-bearing mice and lung cancer cells, and preliminarily explore the potential mechanism(s).

## 2. Results and Discussion

### 2.1. Identification of Chemical Components

**Figure 1:**
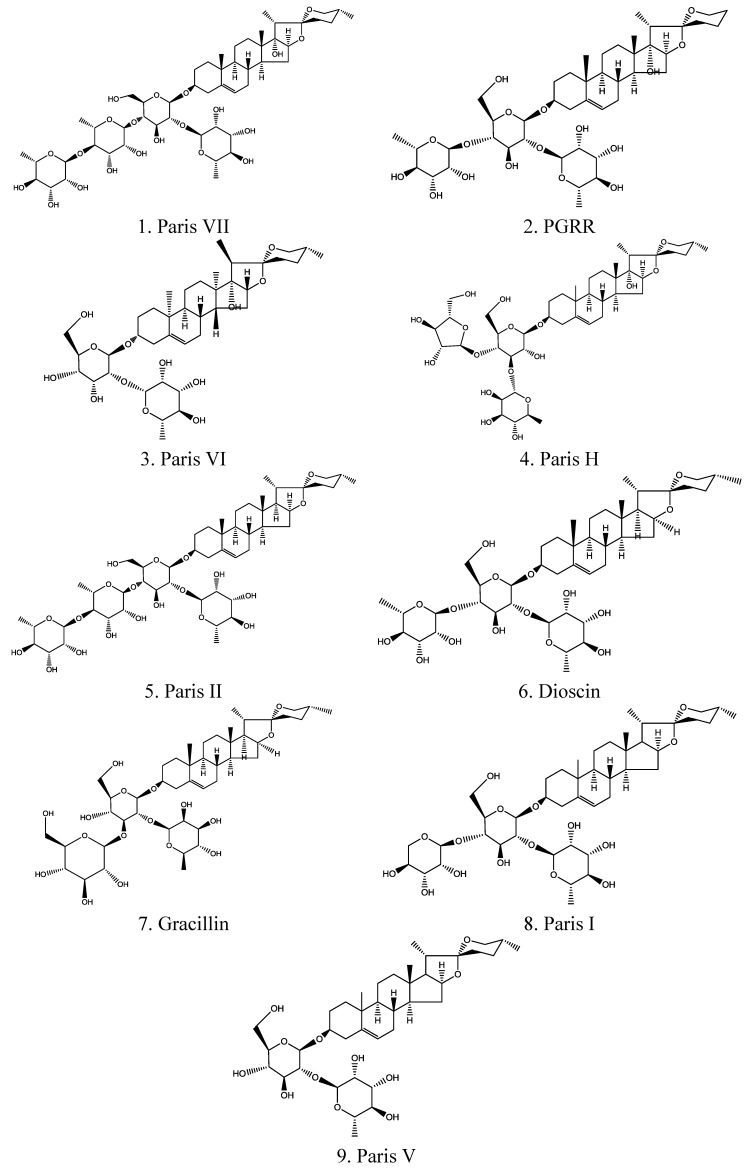
The chemical structure of nine steroidal saponins (PRS) including Paris VII, PGRR, Paris VI, Paris H, Paris II, Dioscin, Gracillin, Paris I and Paris V.

Steroidal saponins were the main compounds of PRS and they have been confirmed as contributors to the inhibition of tumor growth [13]. After being extracted with methanol and *n*-butanol, these main steroidal saponin compounds were analyzed and identified by high performance liquid chromatography-diode array detection (HPLC-DAD) and liquid chromatography-electrospray ionization-tandem mass spectrometry (LC-ESI-MS/MS). As shown in Figure 1 and Table 1, these compounds were identified as Paris VII (11.07%), PGRR (3.82%), Paris VI (21.40%), Paris H (5.04%), Paris II (4.85%), Dioscin (5.16%), Gracillin (2.99%), Paris I (17.29%) and Paris V (19.33%). The retention times of these compounds were 12.774, 13.390, 14.248, 15.122, 24.103, 25.562, 26.125, 27.352, 29.786 min. respectively.

**Figure 2:**
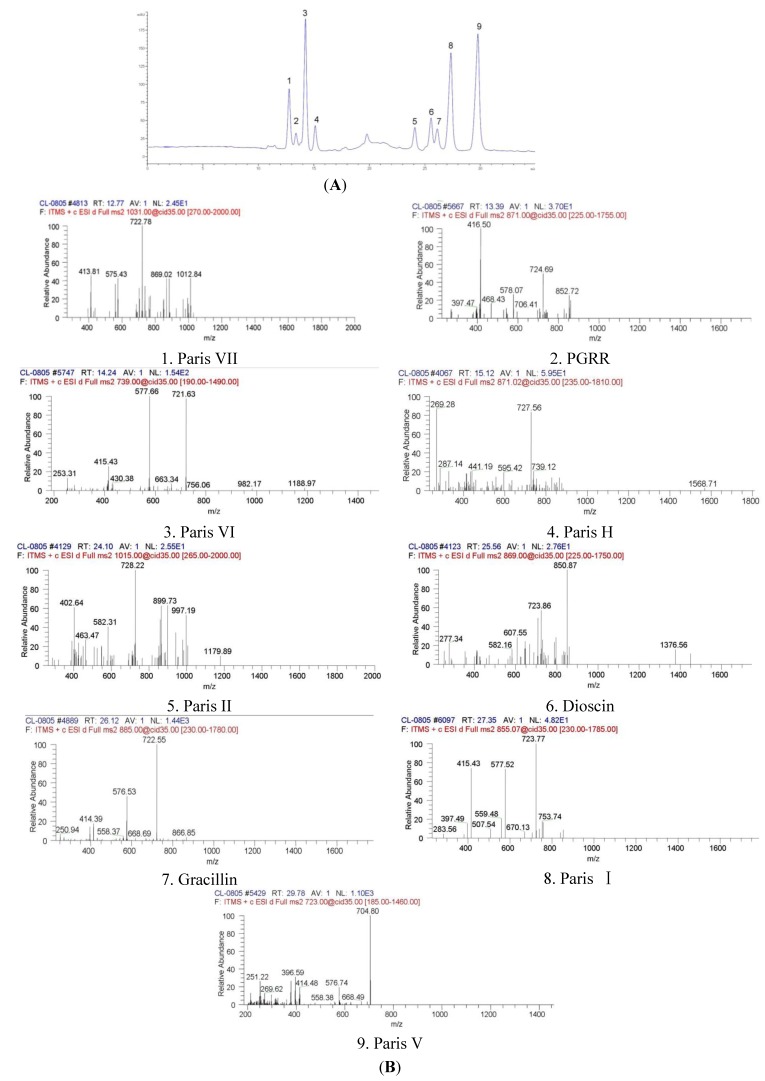
The HPLC chromatogram (**A**) and LC-ESI-MS/MS spectra of PRS (**B**).

Our established HPLC-DAD and HPLC-ESI-MS/MS method (Figure 2) can simultaneously identify the multiple bioactive components in PRS. It is meaningfully helpful in better understanding on the chemical information and underlying mechanisms of PRS’s therapeutic effects.

**Table 1:** The retention time, UVmax and MS ion fragment information of PRS using HPLC-DAD and LC-ESI/MS/MS.

No.	Compound	Chemical nomenclature	T_R_ (min)	(+)ESI-MS m/z	UVmax (nm)	(+)ESI-MS m/z (ion fragments)	Formula
**1**	Paris VII	25(*R*) Pennogenin-3-*O*-*α*-*L*-rhamnopyranosyl (1→4) -*α*-*L*-rhamnopyranosyl (1→4)-[*α*-*L*-rhamnopyranosyl (1→2)]- *β*-*D*-glucopyranoside	12.774	1031.18	200, 280	1012.84, 869.02, 722.78, 575.43, 413.81	C_51_H_82_O_21_
**2**	PGRR	25(R)-5-en-spirost-3β, 17*α*-diol-3-*O*-*α*-*L*-rhamnopyranosyl (1→2)[*α*-*L*-rhamnopyranosyl(1→4)]-*β*-*D*-glucopyranoside	13.390	871.02	205, 280	852.72, 724.69, 578.07, 416.50	C_44_H_70_O_17_
**3**	Paris VI	25(*R*) pennogenin-3-*O*-*α*-*L*- rhamnopyranosyl (1→2) –*β*-*D*-glucopyranoside	14.248	738.90	200, 285	721.63, 577.66, 415.43	C_39_H_62_O_13_
**4**	Paris H	25(*R*) pennogenin-3-*O*-*α*-*L*-arabinofuranosyl(1→4) [*α*-*L*-rhamnopyranosyl (1→2)] -*β*-*D*-glucopyranoside	15.122	871.02	203	727.56, 595.42, 441.19, 287.14	C_44_H_70_O_17_
**5**	Paris II	25(*R*) diosgenin-3-*O*-*α*-*L*-rhamnopyranosyl (1→4) -*α*-*L*-rhamnopyranosyl (1→4)-[*α*-*L*-rhamnopyranosyl (1→2)]- *β*-*D*-glucopyranoside	24.103	1015.18	200	997.19, 899.73, 728.22, 582.31, 402.64	C_51_H_82_O_20_
**6**	Dioscin	25(*R*) diosgenin bis-*α*-*L* -rhamnopyranosyl)-(1→2 and 1→4)- *β*-*D*-glucopyranoside	25.562	869.04	205	850.87, 723.86, 607.55, 277.34	C_45_H_72_O_16_
**7**	Gracillin	25(*R*) diosgenin bis-*α*-*L* -rhamnopyranosyl)-(1→2 and 1→3)-* β*-*D*-glucopyranoside	26.125	885.04	200	722.55, 576.53, 414.39	C_45_H_72_O_17_
**8**	Paris I	25(*R*) diosgenin-3-*O*-*α*-*L*-arabinofuranosyl(1→4) -[*α*-*L*-rhamnopyranosyl (1→2)] –*β*-*D*-glucopyranoside	27.352	855.07	200, 280	723.77, 577.52, 415.43	C_44_H_70_O_16_
**9**	Paris V	25(*R*) diosgenin-3-*O*-*α*-*L*- rhamnopyranosyl (1→2) –*β*-*D*-glucopyranoside	29.786	722.90	200	704.80, 576.74, 396.59, 251.22	C_39_H_62_O_12_

### 2.2. Effect of PRS on Tumor Growth, Tumor Volume, Tumor Weight in C57BL/6 Mice

**Figure 3:**
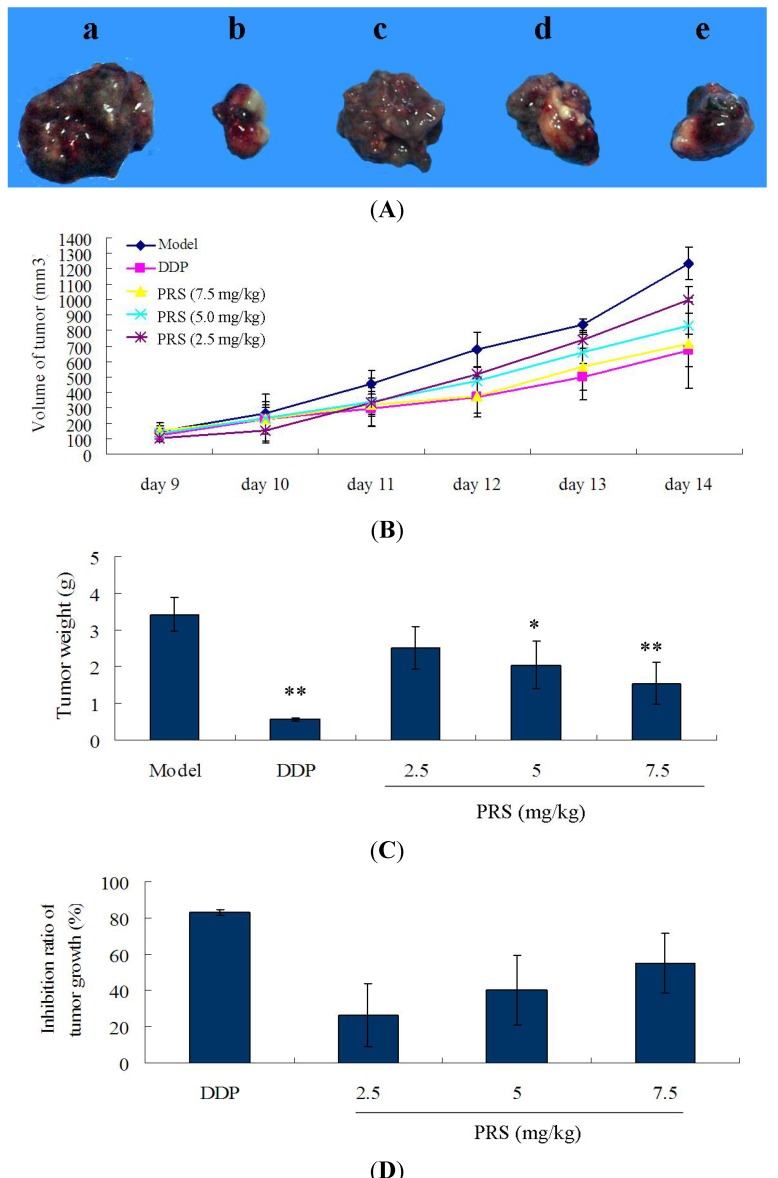
Effect of PPSC on tumor volume (**A** and **B**), tumor weight (**C**) and tumor growth (**D**) in lewis-bearing C57BL/6 mice. These mice were injected with 0.2 mL Lewis cells (10^7^ cells/mL) and administered orally by PRS (2.5, 5.0 and 7.5 mg/kg) from 2nd day to 14th day. This experiment was repeated for three times and at least 5–6 mice for each. (*n =* 15−18). Data are expressed as means ± SD. * *p* < 0.05, ** *p* < 0.01, PRS or DDP *vs*. model.

The treatment of PRS was started on the 2nd day after the ice were injected with tumor cells. After being induced for 8 days, the tumor blocks in right anterior limb could be touched. The tumor volumes of mice were recorded, and the acceleration curve is shown in Figure 3B. From the 11th day to 14th day, the tumor volume of model groups was bigger than that of PRS-treated group and positive cisplatin (DDP) group. The tumor volume in 7.5 mg/kg PRS group was lower than that in 2.5 mg/kg PRS group. As depicted in Figure 3A,C, a significant decrease of the tumor weight in PRS groups were observed in a dose-dependent manner, especially group d and e as compared with model (*p* < 0.05, *p* < 0.01) (Figure 3A). The rates of tumor inhibition were increased significantly by PRS in a dose-dependent manner (26 ± 17% for 2.5 mg/kg; 40 ± 18% for 5.0 mg/kg; 54 ± 16% for 7.5 mg/kg, Figure 3D). All the results above showed that PRS could inhibit the growth of tumor in Lewis lung carcinoma cells-bearing C57BL/6 mice. The study of Yan *et al*. showed that the compounds of PRS significantly inhibited tumor growth in T739 mice bearing LA795 lung adenocarcinoma [14]. In addition, PRS could also inhibit the migration in murine lung adenocarcinoma, both *in vitro* and *in vivo* [9]. These results suggested that PRS might be beneficial for the inhibition of PRS on tumor growth of NSCLC.

### 2.3. Immunomodulatory Effects of PRS on Tumor-Bearing C57BL/6 Mice

**Figure 4:**
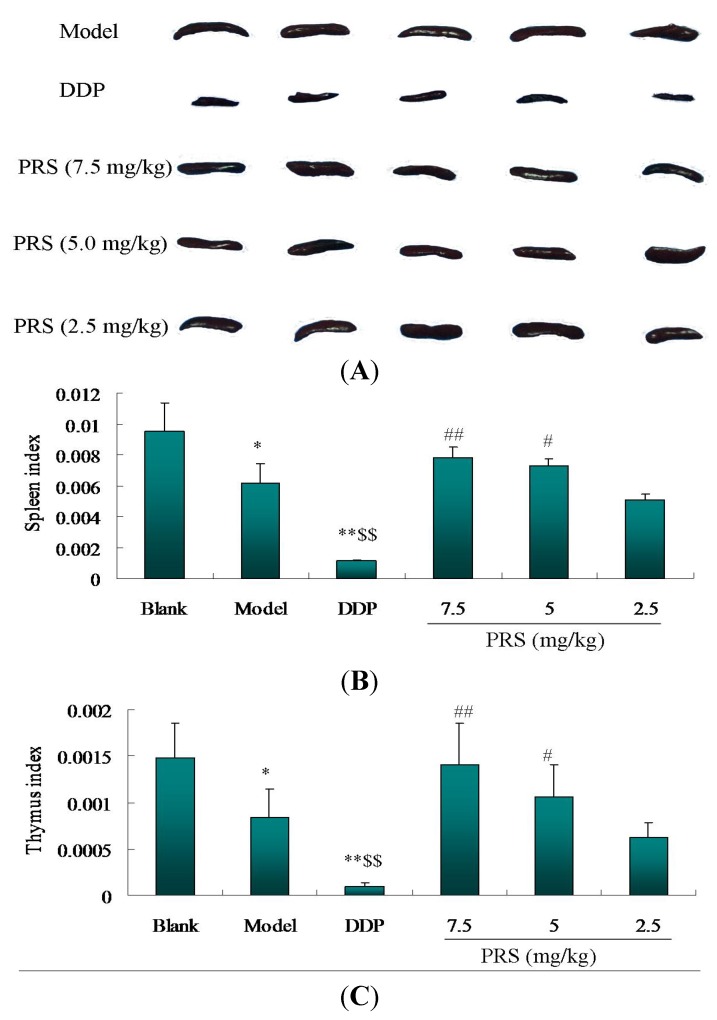
Effect of PRS on spleen index and thymus index in lewis tumor-bearing C57BL/6 mice. After being sacrificed, the spleen (**A**) and thymus of mice were taken for weight. The spleen weight index (**B**) and the thymus index (**C**) were evaluated according to the formula in Section 3.5. The data are taken for three individual experiment and expressed as means ± SD (*n =* 15−18). * *p* < 0.05, ** *p* < 0.01, model or DDP *vs*. blank control; ^$$^ *p* < 0.01, DDP *vs*. model; ^#^ *p* < 0.05, ^##^ *p* < 0.01, PRS *vs*. DDP.

The decrease of immune function contributes to the development and progression of lung cancer [15]. There is accumulating evidence highlighting the immunological responses play an integral role in lung cancer [16]. Spleen and thymus are two immune organs which strengthen the immune surveillance in order to phagocytose and remove tumor cells and dead tissue from the circulating blood [17]. The decrease of the relative spleen and thymus weight has been regarded as the most sensitive indicator of immunosuppression. Spleen index and thymus index, two immune parameters, are related closely to the immune function. In tumor-bearing mice, these two indexes were used usually for the evaluation of immune function. As shown in Figure 4A–C, the spleen index and thymus index of the model group were much lower than those of the blank control group (*p* < 0.01).

However, PRS (5.0 and 7.5 mg/kg) significantly increased the spleen index and the thymus index of tumor-bearing mice. There was no difference in spleen weight index and the thymus index between low-dose PRS group and model group. The spleen and thymus indexes in DDP-treated group were lower than that of the blank group (*p* < 0.05). Although the rate of tumor growth inhibition in the DDP group had an obvious advantage over the other groups, the spleen index and thymus index were lower than that of the PRS groups in tumor-bearing C57BL/6 mice. Our data showed that PRS alleviated the decreased sizes of the spleen and thymus in tumor-bearing C57BL/6 mice. Accumulating evidences support the fact that immunostimulation is a complex biological phenomenon ocurring through multiple mechanisms [17]. These results led the researchers to speculate that the anti-lung cancer activity of PRS might be related to immune function enhancement.

### 2.4. PRS Decreased TNF-α, IL-8 and IL-10 Levels of Serum in C57BL/6 Mice

Clinical studies show that inflammation-related factors are associated with the risk of lung cancer [18]. Multiple cytokines were secreted, functionalized and regulated by a wide variety of cell populations and then resulted in a network of pro-inflammatory cytokines. As can be seen in Table 2, there is a severe increase of TNF-α, IL-8 and IL-10 as compared to uninduced normal mice. The oral administration of PRS (5.0 and 7.5 mg/kg) could significantly down-regulate the levels of these cytokines related to the inflammation response (*p* < 0.05, *p* < 0.01). Although no significant differences were observed between low-dose PRS and model mice (2.5 mg/kg), the mean values of TNF-α, IL-8 and IL-10 were reduced. In the serum of NSCLC patients, the levels of IL-8 and IL-10 were higher than that of normal people [19]. TNF-α induced NF-kappa B alpha kinase activation, leading to the regulation of carcinogenesis and inflammation in human NSCLC [20]. The saponins of PPSCFH has been found to hold immunostimulating properties for RAW 264.7 macrophage cells [21]. However, the underlying mechanism of these effects remains unclear. In particular, it is not completely clear the mechanism of PRS on regulating these cytokines. In spite of this, our findings suggested that PRS could down-regulate inflammatory cytokines in Lewis tumor-bearing mice through the immunomodulatory function.

**Table 2:** Effect of PRS on the levels of inflammatory factors in Lewis tumor-bearing mice (*n* = 6).

Groups	Dose (mg/kg/day)	TNF-α (pg/mL)	IL-8 (pg/mL)	IL-10 (pg/mL)
Blank control	−	71.1 ± 8.2	45.8 ± 8.4	44.7 ± 5.7
Model	−	106.2 ± 6.7 **	85.4 ± 10.3 **	77.3 ± 6.8 **
DDP	20	91.7 ± 9.3 ^#^	58.5 ± 5.5 ^#^	53.9 ± 4.2 ^#^
PRS	7.5	74.8 ± 11.5 ^##^	64.7 ± 6.8 ^#^	59.0 ± 8.5 ^#^
	5	77.3 ± 5.3 ^#^	71.7 ± 7.2 ^#^	68.3 ± 11.4
	2.5	83.9 ± 6.6	82.6 ± 4.6	76.5 ± 10.6
Standard curve	−	Y = 0.0006x + 0.0304 (R^2^ = 0.9824)	Y = 0.0037x + 0.1800 (R^2^ = 0.9110)	Y = 0.0001x + 0.0960 (R^2^ = 0.9977)

** *p* < 0.01, model *vs*. blank control; ^##^ *p* < 0.05, *p* < 0.01, PRS *vs*. model, DDP *vs*. model.

### 2.5. Effect of PRS on the Proliferation of Lewis Cells and A549 Cells

**Figure 5:**
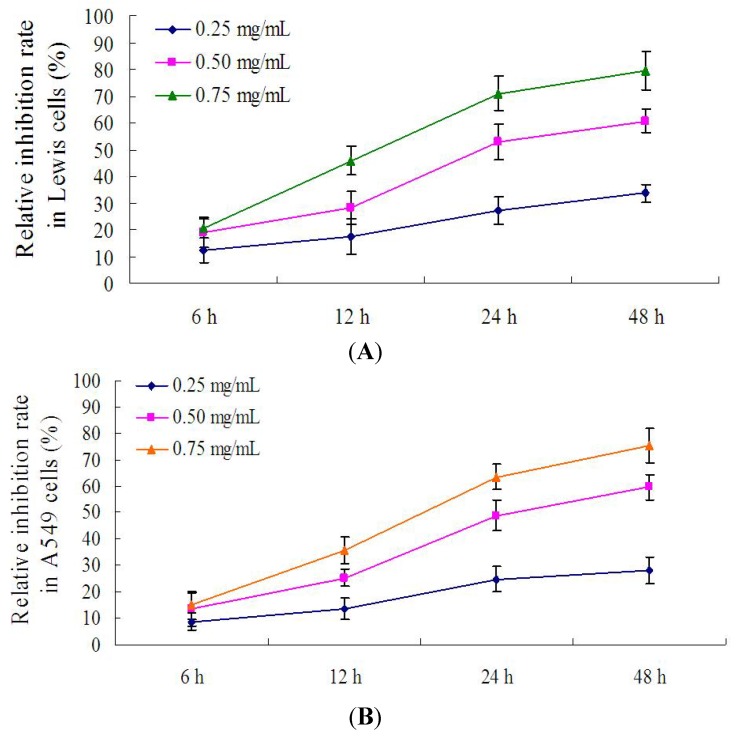
The inhibition of PRS on the proliferation of Lewis cells and A549 cells at different time points. Lewis cells and A549 cells were treated respectively with PRS (0.25, 0.50 and 0.75 mg/mL) at 6, 12, 24 and 48 h. The relative inhibition rate was calculated according to the OD values by MTT assay (*n* = 9).

In order to screen the optimal incubation time, different time points including 6, 12, 24 and 48 h were chosen to evaluate the inhibition of PRS on the proliferation of lewis cells and A549 cells. As shown in Figure 5A,B, the rate of inhibition of PRS (0.25, 0.50 and 0.75 mg/mL) was increased sharply from 6 h to 24 h. However, the increase of inhibition rate raised slowly from 24 h to 48 h. The results showed that the inhibition rates of PRS in Lewis cells and A549 cells are close to those at 48 h. These findings demonstrated that 24 h incubation time might be the acceptable time for further experiments.

### 2.6. PRS induced Apoptosis of A549 Cells

**Figure 6:**
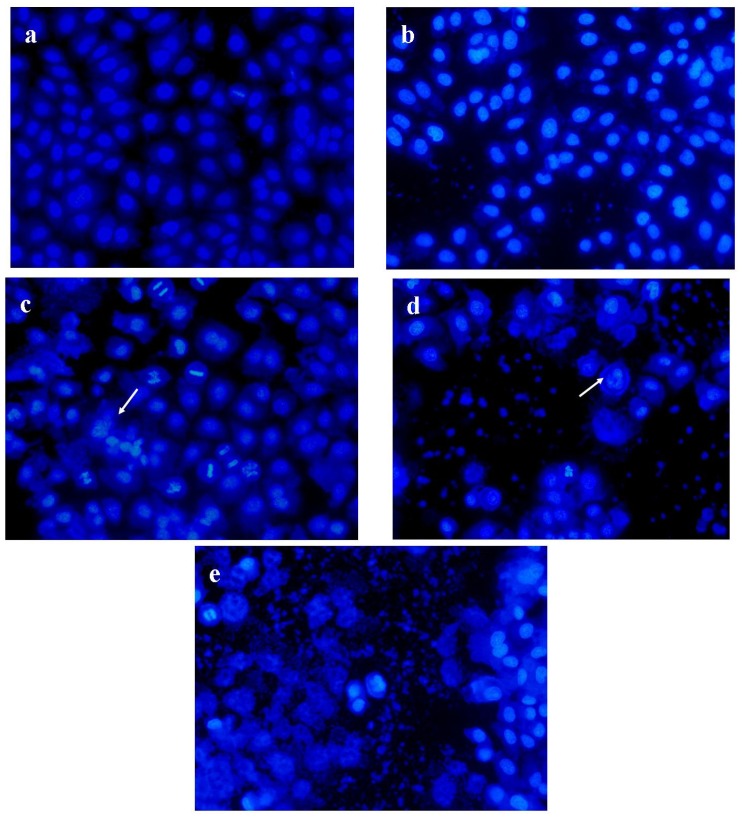
The induction of PRS on nuclei/chromatin in A549 cells by Hoechst 33342 staining. (**a**) blank control; (**b**) PRS 0.25 mg/mL; (**c**) PRS 0.50 mg/mL; (**d**) PRS 0.75 mg/mL; (**e**) 10 μg/mL cisplatin. A549 cells were exposed to the mentioned PRS for 24 h. Fluorescence images of Hoechst 33342 stained A549 cells were obtained. Arrowheads point to the cells with abnormal nuclei, indicating fragmentation of nuclei/chromatin. Cisplatin of 10 μg/mL was chosen as a positive control. Magnification 200 ×.

In order to evaluate the effect of PRS on tumor cells, Hoechst 33342 staining was performed to evaluate the apoptosis on A549 cells. The type of Lewis lung carcinoma cell line was from a murine source. It was more suitable for mice *in vivo* while A549 cells are not. However, the A549 cell line was used for the further evaluation *in vitro* due to the significance of this human cell line. As depicted in Figure 6, A549 cells in the blank control were observed as round-shaped nuclei with homogeneous fluorescence intensity. Most nuclei of cells in blank control exhibited regular contours and were round and large. After being treated with PRS, nuclear changes of A549 cells with DNA condensation and fragmentations of chromatin were induced in a concentration-dependent manner. Our results indicated that PRS could induce apoptosis to lead to cell death in A549 cells.

**Figure 7:**
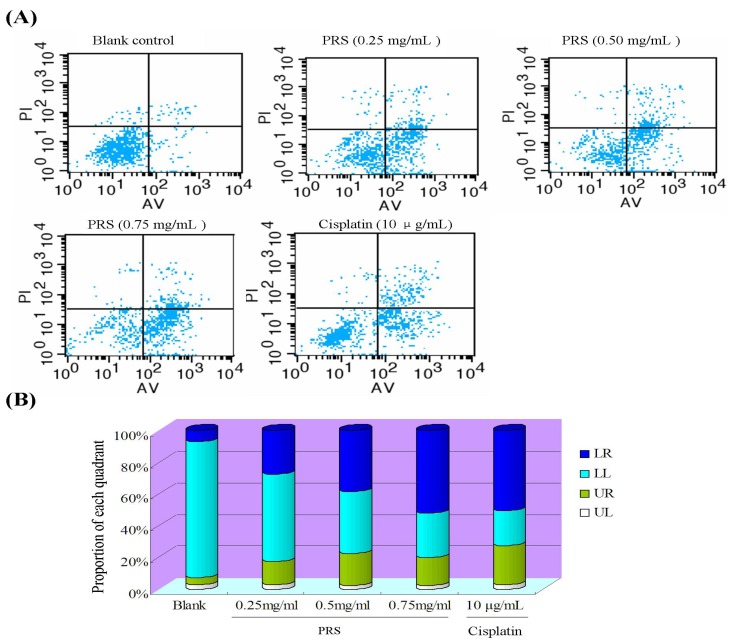
Effect of PRS on annexin V/PI-stained apoptosis in A549 cell by flow cytometry. A549 cells were exposed to PRS (0.25, 0.50 and 0.75 mg/mL) and positive control cisplatin of (10 μg/mL) for 24 h. (**A**) annexin V/PI images; (**B**) the ratio of different quadrants.

Annexin V/propidium iodide (PI) by flow cytometry was performed to further evaluate the induction apoptosis of PRS in A549 cells [22]. After being stained with annexin V/PI, the apoptosis of A549 cells were analyzed with flow cytometry (Figure 7).

### 2.7. PRS Reduced ROS Generation of A549 Cells

Oxidative damage has been shown to play an important role in the onset and progression of lung cancer [23]. The imbalance of the oxidation system causes excessive production of ROS when cells undergo chemical or environmental stress [24].

**Figure 8:**
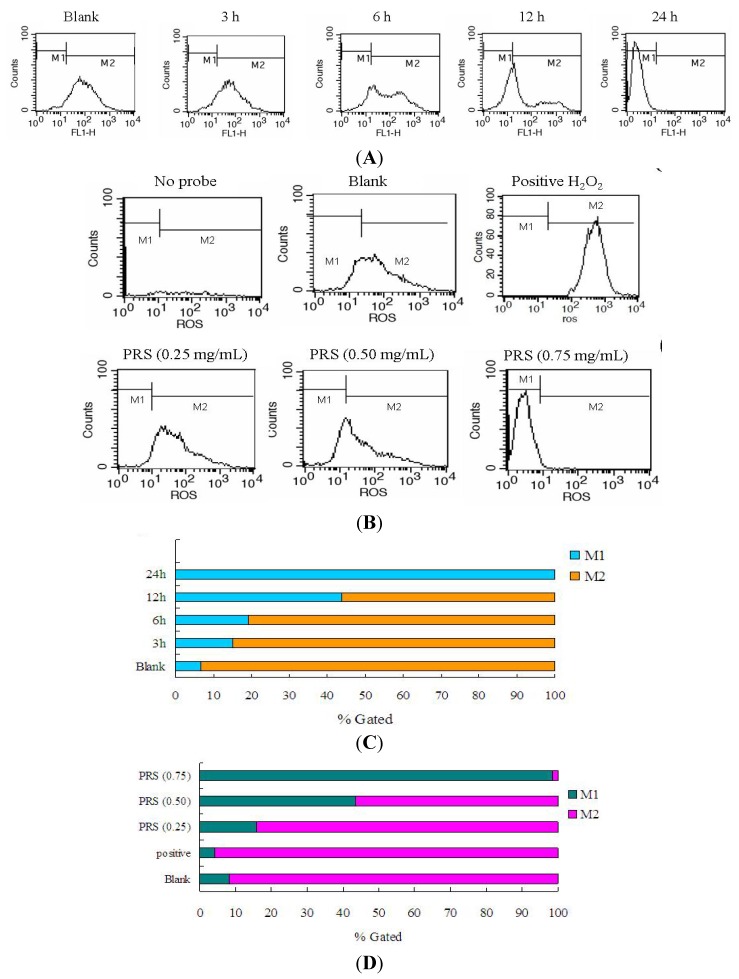
Reduction of PRS on ROS generation in A549 cells. Cells were induced in absence or presence of PRS (0.5 mg/mL) for 3, 6, 12 or 24 h (**A** and **C**) and then ROS production was assessed by flow cytometry with a fluorescence probe 2',7'-dichlorodihydrofluorescein diacetate (DCFH-DA) staining. Furthermore, A549 cells were treated with PRS (0.25, 0.5, 0.75 mg/mL) for 24 h (**B** and **D**). In this study, 100 μM H_2_O_2_ was chosen as a positive control. The significant increase in ROS was calculated the counts in the M2 region.

The accumulation of ROS has been regarded as one of the causative factors leading to cell apoptosis or inflammation [25]. Both clinical and animal experiments have demonstrated that ROS level was significantly increased in lung cancer patients and tumor-bearing mice [24,26]. Therefore, reducing oxidative damage is useful for ameliorating inflammation response and apoptosis. In this study, we examined the ROS level in control or PRS-treated A549 cells by staining with 2',7'-dichlorodihydrofluorescein diacetate (DCFH-DA) dye. A549 cells were treated with 0.5 mg/mL PRS for 3, 6, 12 or 24 h. As shown in Figure 8A,C, the ROS generation was increased gradually with the lengthen of periods. 24 h was chosen as the optimal incubation time. Figure 8B,D demonstrated that PRS could remarkably reduce intracellular ROS of A549 cells. The over-generation of ROS can cause nuclear DNA damage and then result in cell death, survival, or senescence [27]. Apoptosis of tumor cells can be triggered through two classic pathways: (1) the death receptor-mediated extrinsic pathway and (2) mitochondria-mediated intrinsic pathway. In our study, the ROS level was decreased by PRS, while apoptosis was induced, so we speculate that the induction of PRS on apoptosis may be associated with apoptosis receptor. Our results indicated that PRS might be beneficial in the regulation of the oxidative status of A549 cells.

### 2.8. PRS Decreased the Expressions of Inflammation-Related Proteins

**Figure 9:**
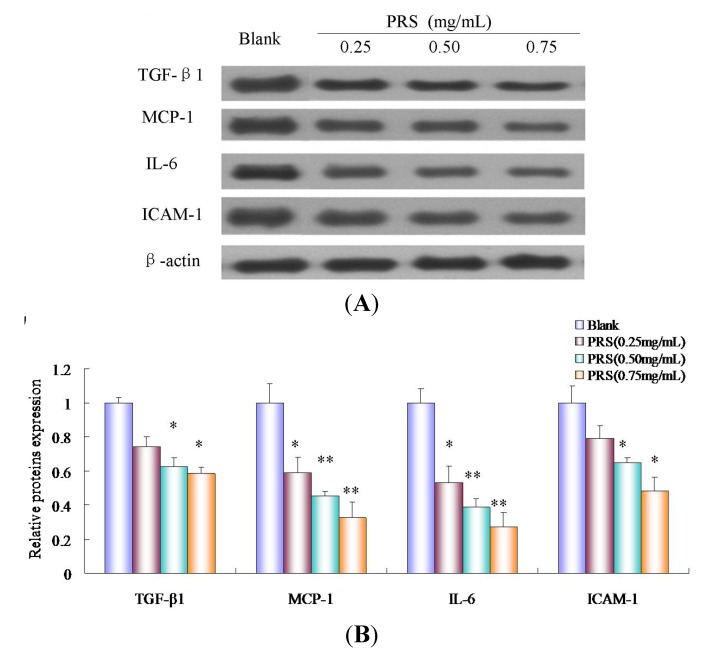
Effect of PRS on the expressions of inflammation-related proteins by Western blotting. A549 cells were incubated in the presence and absence of PRS (0.25, 0.50 and 0.75 mg/mL) for 24 h. Cells were homogenized, lysed, 50 μg of protein samples were submitted to 10% SDS-PAGE and electroblotted. Enhanced chemiluminescence system was used for visualization (**A**) and protein expression was quantified by IPP software. Results are expressed as the ratio of the band volume of PRS to that of blank. The data are expressed as means ± SD (*n = * 6). * *p* < 0.05; ** *p* < 0.01, *vs*. blank.

The effects of PRS on the expressions of cancer-related inflammation cytokines, including transforming growth factor-β1 (TGF-β1), IL-6, intercellular adhesion molecule-1(ICAM-1) and monocyte chemotactic protein 1(MCP-1) in A549 cells, were explored further by western blotting. The expressions of TGF-β1, IL-6, ICAM-1 and MCP-1 were reduced significantly by PRS (0.25, 0.50 and 0.75 mg/mL) in a concentration-dependent manner, compared to blank (Figure 9A,B). These data clearly demonstrated the potent anti-inflammation activity of PRS in A549 cells. It suggested that modulating inflammation cytokines of PRS might play an important role in the proliferation of tumor cells.

Inflammatory processes have been found to be an important mechanism of immunomodulatory and are considered to play an important role in the proliferation of tumor cells. Studies showed that the induction of cell adhesion molecules and proinflammatory cytokines implicated in inflammatory processes during the growth of tumor. The release of proinflammatory cytokines such as MCP-1, IL-6 and TGFβ1, as well as cell adhesion molecule ICAM-1, could lead to an increase of migration ability in A549 cells [28,29,30,31,32]. Lee *et al*. showed that the inflammation in lung tissue could be modulated via ROS-dependent ICAM-1 inhibition [33]. Combined the results of ELISA, our data indicated that PRS treatment could inhibit the growth of tumor or the proliferation of tumor cells through attenuating inflammation responses which were responsible for the observed immunomodulatory effects, at least in part.

## 3. Experimental

### 3.1. General

The dried rhizomes of PPSCFH were purchased from Jiangsu Pharmaceutical Corporation (Nanjing, China) and identified as P*. polyphylla* Smith var. *chinensis* (Franch.) Hara by Prof. Dekang Wu from the Nanjing University of Chinese Medicine. The voucher specimen was deposited in our laboratory (No. ACM 20130314). IL-10, IL-8 and TNF-α ELISA kits were purchased from KeyGEN Biotech. Co., Ltd. (Nanjing, China). ICAM-1, IL-6, TGF-β1 and MCP-1 antibodies were obtained from Wuhan BOSTER Biological Engineering Co., Ltd. (Wuhan, China). ROS kit was obtained from Beyotime Institute of Biotechnology Co.,Ltd. (Jiangsu, China). HPLC-grade acetonitrile was purchased from TEDIA (Farfield, OH, USA). Other reagents were from commercial sources and analytical-grade.

### 3.2. Preparation of PRS Extract

The rhizomes of PPSCFH were dried at 50 °C to a constant weight, ground into powder and then passed through a 40 mesh sieve. The powder (100 g) was weighed and extracted with methanol (500 mL) by refluxing for 1.5 h/time (two times). The two extracts were combined and then the methanol was recovered by rotary evaporation at 60 °C. A crude extract of 6.58 g was obtained. Sequentially, *n*-butanol (50 mL) was added and extracted three times. The *n*-butanol fraction was recovered by rotary evaporation to dryness under reduced pressure. A 3.29 g *n*-butanol fraction was obtained. Finally, the extract was dissolved in HPLC-grade methanol for HPLC-DAD and LC-ESI-MS/MS analysis.

### 3.3. HPLC and LC/MS/MS Analysis

The component analysis was performed on an Agilent 1200 HPLC system (Agilent Technologies, Palo Alto, CA, USA), equipped with a quaternary pump, an automatic sample injector, diode array detector (DAD) and Agilent ChemStation software as the data acquisition system. The separation was performed on a ZORBAX C_18_ column (4.6 × 250 mm, 5 μm). The mobile phase consisted of acetonitrile (A) and 0.1% formic acid was used for the elution of compounds with a flow rate of 1.0 mL/min. The optimization gradient elution process was set at as follows: 0–10 min, 40%A–40%A; 10–15 min, 40%–45% A; 15–25 min, 45%–45% A; 25–60 min, 45%–60% A. Detection wavelength was set at 210 nm. Injection volume was 10 μL and column temperature was maintained at 25 °C.

HPLC-ESI-MS/MS was used to identify the compounds. In this study, a Thermo Fisher Scientific (Bremen, Germany) ion trap mass spectrometer (model LCQ) equipped with an electrospray ionization (ESI) interface was used. Xcalibur version 2.0 controlling software was used for data acquisition and processing. Typical positive ESI-MS conditions were kept as follows: ion spray voltage (−4.5 kV), capillary temperature (300 °C). capillary voltage (3.0 kV), skimmer cone voltage(−20 V); nebulizing gas (N_2_), collision gas (He); sheath gas (N_2_, 40 a.u), auxiliary gas (N_2_, 10 a.u.), MS full-scan range (100–2,000).

### 3.4. Cell Culture

Lewis mice cell line and human lung adenocarcinoma A549 cell line were purchased from ATCC. Lewis cells were incubated in DMEM medium, supplemented with 10% fetal bovine serum (FBS), 100 U/mL of penicillin, 100 µg/mL of streptomycin. Cells were maintained in a humidified incubator at 5% CO_2_ and 37 °C. The medium was renewed every 2 days. These cells were detached by 0.25% trypsin-0.01% EDTA and used for seeding. A549 cells were cultured in 25 cm^2^ flasks with RPMI-1640 medium supplemented with 0.1 M L-glutamine, 10% FBS, 100 U/mL of penicillin, 100 µg/mL of streptomycin. Cells were incubated at 37 °C with 5% CO_2_ in a fully humidified incubator. Cells of passaged 3–4 were used for experiment.

### 3.5. Lewis Tumor-Bearing C57BL/6 Mice and Treatment

Lewis lung carcinoma cells-bearing C57BL/6 mice were prepared for the evaluation of PRS *in vivo* according to our previous method [34]. Male C57BL/6 mice (18–20 g) of 6–8 weeks age were provided by Shanghai SLAC Laboratory Animal Co., Ltd. All animal procedures were approved by the Provision and General Recommendation of Chinese Experimental Animals Administration Legislation. Briefly, mice were maintained under standard environmental conditions: temperature of 25 °C ± 1 °C; relative humidity of 50% ± 5%. Mice were fed with a standard diet and water *ad libitum*. Lewis cells were harvested from flasks by 0.25% trypsin-0.01% EDTA and prepared to a concentration of 1 × 10^7^ cells/mL. The cell suspension of 0.2 mL were inoculated subcutaneously into the armpit of right anterior superior limbs of C57BL/6 mice. Next day, mice were divided into non-tumor cell group, tumor model group, PRS groups (2.5, 5.0 and 7.5 mg/kg), and positive group DDP (20 mg/kg) (5–6 mice for each group/experiment). Mice in non-tumor cell group and tumor model group were given with 0.2 mL 0.9% NaCl. Mice in PRS groups were administered orally by dfferent concentrations PRS of 0.2 mL/day whereas 0.2 mL/day DDP for positive control through intraperitoneal injection. The treatment lasted for 14 days. The volumes of tumor were measured daily from the 9th day. At the end of treatment, mice were sacrificed by cervical dislocation and then the solid tumors, spleens and thymuses were then excised and weighed. Blood samples were taken through orbital venous. The inhibitive rate of tumor was calculated as follows: inhibitive rate = [1 − (tumor weight in each drug treatment group/average tumor weight of model group)] × 100%. The spleen and thymus indexs were evaluated according to the following formula: spleen or thymus weight (g)/body weight (g). Experiments were repeated three times (15–18 mice in each group).

### 3.6. ELISA Assay for Inflammatory Cytokines

Blood samples were centrifuged with 4,000 × g for 15 min and the serum was taken for the determination of inflammatory cytokines. ELISA kits were used to determine the serum levels of cytokines according to the manufacturer’s protocols. The optical density of samples were determined with microplate reader at 450 nm wavelength. The contents of inflammatory cytokines were calculated according to the standard curve.

### 3.7. Hoechst 33342 Staining

Hoechst 33342 staining was conducted as described previously [35]. In brief, cells (1 × 10^6^) were exposed to PRS (0.25, 0.50 and 0.75 mg/mL) for 24 h and then rinsed three times in cold PBS and fixed with 4% fresh formaldehyde at 4 °C for 10 min. Cells were co-incubated with 5 μg/mL Hoechst 33342 stain and maintained at 37 °C for 10 min in the dark. After staining, cells were observed and photographed under an AXIO Scope1 fluorescence microscope (Axiocam, Carl Zeiss, Germany) with an excitation of 350 nm and an emission of 465 nm [29].

### 3.8. Annexin-V/PI Double-Staining

Effect of PRS on apoptosis of A549 cells was measured by flow cytometry using Annexin V/PI double staining. A549 cell suspensions (3 × 10^5^ cells/well) were seeded into 600 mm-diameter culture dishes for apoptosis analysis. After being treated with mentioned concentration PRS (0.25, 0.5 and 0.75 mg/mL) for 24 h. Cells were harvested and washed with ice-cold PBS twice. After washing, cells were resuspended in 300 mL binding buffer containing Annexin V (3 µL) and PI (3 µL) for 15 min at room temperature in the dark. Finally, cell apoptosis was quantified by FACScan flow cytometry (Becton Dickinson, Franklin Lakes, NJ, USA). In this measurement, at least 10,000 cells of each sample were analyzed. The apoptosis rate was calculated according to the following formula: Apoptosis rate (%) = (number of apoptotic cells)/(number of total cells observed) × 100%.

### 3.9. Reactive Oxygen Species (ROS) Assay

The ROS generation was determined as described previously [36]. Briefly, A549 cells were exposed to drugs and then harvested. After being washed once with ice-cold PBS of 3 mL, cells were incubated with 50 μmol/L DCFH-DA at 37 °C for 20 min in the dark. Intracellular ROS generation was measured FACSCalibur flow cytometer with an 488 nm excitation and 530 nm emission wavelength. The number of each sample was at least 10,000 cells. CellQuest software was used to quantity the fluorescence intensity of DCF. The experiment was performed for three times [30].

### 3.10. Western Blotting Analysis

Western blotting analysis was performed according to the method of Satoru *et al*. with a slight modification [37]. After stimulation, A549 cells were lysed with cold Triton buffer (1% Triton X-100, 150 mM NaCl, 1 mM EDTA, 20 mM Tris-HCl, 5 μg/mL pepstatin A, 2 mM diisopropylfluorophosphate, and 1 mM phenylmethylsulfonylfluoride). The lysates were centrifuged at 12,000 × g for 15 min, and then supernatant was taken for quantity of protein concentrations. An equal amount of proteins (50 μg) was separated by 10% SDS-PAGE and then transferred to a nitrocellulose membrane. After being incubated with 1% BSA in tris-buffered saline Tween-20 (TBST), membrane was incubated with anti-human IL-6 (1:200 dilution), TGF-β1(1:200 dilution), ICAM-1(1:200 dilution) and MCP-1 (1:200 dilution) overnight. Then, membrane was washed thrice, and incubated with the peroxidase-conjugated secondary antibody (1:1000 dilution). Finally, the bands in the membrane were visualized with enhanced chemiluminescence system (USA) and photographed by an ECL minicamera. The quantify of brands was analyzed by Image pro plus (IPP) software. β-actin was used for loading control.

### 3.11. Statistical Analysis

All data were presented as means ± standard deviation (SD). Statistical analysis was performed by SPSS 16.0 software with one-way ANOVA. Significant difference was measured within the groups. The value of *p* less than 0.05 was considered to be a statistically significant difference.

## 4. Conclusions

In conclusion, this study demonstrated for the first time that the inhibition of tumor growth on experimental lung cancer and its attenuation by the steroidal saponins of PRS might occur via attenuation of the inflammation response, induction of apoptosis in the tumor cells and reduction of the accumulation of ROS. The present study has shown that PRS appears to have multiple actions relevant to inhibit tumor growth. Our *in vitro* and *in vivo* results with PRS suggest that it may constitute a promising intervention agent in the prevention and treatment of lung cancer.

## References

[1] Carpagnano G.E., Palladino G.P., Lacedonia D., Koutelou A., Orlando S. (2011). Foschino-Barbaro MP. Neutrophilic airways inflammation in lung cancer: The role of exhaled LTB-4 and IL-8. BMC Cancer.

[2] Umekawa K., Kimura T., Kudoh S., Suzumura T., Oka T., Nagata M., Mitsuoka S., Matsuura K., Nakai T., Yoshimura N. (2013). Plasma RANTES, IL-10 and IL-8 levels in non-small-cell lung cancer patients treated with EGFR-TKIs. BMC. Res. Notes.

[3] Lee J.G., Cho B.C., Bae M.K., Lee C.Y., Park I.K., Kim D.J., Ahn S.V., Chung K.Y. (2009). Preoperative C-reactive protein levels are associated with tumor size and lymphovascular invasion in resected non-small cell lung cancer. Lung Cancer.

[4] Fukuyama T., Ichiki Y., Yamada S., Shigematsu Y., Baba T., Nagata Y., Mizukami M., Sugaya M., Takenoyama M., Hanagiri T. (2007). Cytokine production of lung cancer cell lines: Correlation between their production and the inflammatory/immunological responses both *in vivo* and in vitro. Cancer Sci..

[5] Kang L.P., Liu Y.X., Eichhorn T., Dapat E., Yu H.S., Zhao Y., Xiong C.Q., Liu C., Efferth T., Ma B.P. (2012). Polyhydroxylated steroidal glycosides from Paris polyphylla. J. Nat. Prod..

[6] Li F.R., Jiao P., Yao S.T., Sang H., Qin S.C., Zhang W., Zhang Y.B., Gao L.L. (2012). Paris polyphylla Smith extract induces apoptosis and activates cancer suppressor gene connexin26 expression. Asian Pac. J. Cancer Prev..

[7] Zhao Y., Kang L.P., Liu Y.X., Liang Y.G., Tan D.W., Yu Z.Y., Cong Y.W., Ma B.P. (2009). Steroidal saponins from the rhizome of Paris polyphylla and their cytotoxic activities. Planta Med..

[8] Sun J., Liu B.R., Hu W.J., Yu L.X., Qian X.P. (2007). *In vitro* anticancer activity of aqueous extracts and ethanol extracts of fifteen traditional Chinese medicines on human digestive tumor cell lines. Phytother. Res..

[9] Man S.L., Gao W.Y, Zhang Y.J., Ma C.Y., Yang L., Li Y.W. (2011). Paridis saponins inhibiting carcinoma growth and metastasis *in vitro* and *in vivo*. Arch. Pharm. Res..

[10] Kong M., Fan J., Dong A., Cheng H., Xu R. (2010). Effects of polyphyllin I on growth inhibition of human non-small lung cancer cells and in xenograft. Acta Biochim. Biophys. Sin..

[11] Ma D.D., Lu H.X., Xu L.S., Xiao W. (2009). Polyphyllin D exerts potent anti-tumour effects on Lewis cancer cells under hypoxic conditions. J. Int. Med. Res..

[12] Siu F.M., Ma D.L., Cheung Y.W., Lok C.N., Yan K., Yang Z., Yang M., Xu S., Ko B.C., He Q.Y. (2008). Proteomic and transcriptomic study on the action of a cytotoxic saponin (Polyphyllin D): Induction of endoplasmic reticulum stress and mitochondria-mediated apoptotic pathways. Proteomics.

[13] Wu X., Wang L., Wang H., Dai Y., Ye W.C., Li Y.L. (2012). Steroidal saponins from Paris polyphylla var. yunnanensis. Phytochemistry.

[14] Yan L.L., Zhang Y.J., Gao W.Y., Man S.L., Wang Y. (2009). *In vitro* and *in vivo* anticancer activity of steroid saponins of Paris polyphylla var. yunnanensis. Exp. Oncol..

[15] Jadus M.R., Natividad J., Mai A., Ouyang Y., Lambrecht N., Szabo S., Ge L., Hoa N., Dacosta-Iyer M.G. (2012). Lung cancer: A classic example of tumor escape and progression while providing opportunities for immunological intervention. Clin. Dev. Immunol..

[16] Ronan J.K., James L.G., Giuseppe G. (2010). Immunotherapy for non-small cell lung cancer. Clin. Lung Cancer.

[17] Aikemu A., Umar A., Yusup A., Upur H., Berké B., Bégaud B., Moore N. (2012). Immunomodulatory and antitumour effects of abnormal Savda Munziq on S180 tumour-bearing mice. BMC Complement. Altern. Med..

[18] Bai L., Yu H., Wang H., Su H., Zhao J., Zhao Y. (2013). Genetic single-nucleotide polymorphisms of inflammation-related factors associated with risk of lung cancer. Med. Oncol..

[19] Kaminska J., Kowalska M., Kotowicz B., Fuksiewicz M., Glogowski M., Wojcik E., Chechlinska M., Steffen J. (2006). Pretreatment serum levels of cytokines and cytokine receptors in patients with non-small cell lung cancer, and correlations with clinicopathological features and prognosis. M-CSF—An independent prognostic factor. Oncology.

[20] Shishodia S., Koul D., Aggarwal B.B. (2004). Cyclooxygenase (COX)-2 inhibitor celecoxib abrogates TNF-induced NF-kappa B activation through inhibition of activation of I kappa B alpha kinase and Akt in human non-small cell lung carcinoma: correlation with suppression of COX-2 synthesis. J. Immunol..

[21] Zhang X.F., Cui Y., Huang J.J., Zhang Y.Z., Nie Z., Wang L.F., Yan B.Z., Tang Y.L., Liu Y. (2007). Immuno-stimulating properties of diosgenyl saponins isolated from Paris polyphylla. Bioorg. Med. Chem. Lett..

[22] Feng L., Jia X., Zhu M., Chen Y., Shi F. (2010). Chemoprevention by Prunella vulgaris L. extract of non-small cell lung cancer via promoting apoptosis and regulating the cell cycle. Asian Pac. J. Cancer Prev..

[23] Suchaoin W., Chanvorachote P. (2012). Caveolin-1 attenuates hydrogen peroxide-induced oxidative damage to lung carcinoma cells. Anticancer Res..

[24] Uneri C., Sari M., Bağlam T., Polat S., Yüksel M. (2006). Effects of vitamin E on cigarette smoke induced oxidative damage in larynx and lung. Laryngoscope.

[25] Demetriou C.A., Raaschou-Nielsen O., Loft S., Møller P., Vermeulen R., Palli D., Chadeau-Hyam M., Xun W.W., Vineis P. (2012). Biomarkers of ambient air pollution and lung cancer: A systematic review. Occup. Environ. Med..

[26] Fortunati N., Manti R., Birocco N., Pugliese M., Brignardello E., Ciuffreda L., Catalano M.G., Aragno M., Boccuzzi G. (2007). Pro-inflammatory cytokines and oxidative stress/antioxidant parameters characterize the bio-humoral profile of early cachexia in lung cancer patients. Oncol. Rep..

[27] Panieri E., Gogvadze V., Norberg E., Venkatesh R., Orrenius S., Zhivotovsky B. (2013). Reactive oxygen species generated in different compartments induce cell death, survival, or senescence. Free Radic. Biol. Med..

[28] Kimura C., Hayashi M., Mizuno Y., Oike M. (2013). Endothelium-dependent epithelial-mesenchymal transition of tumor cells: Exclusive roles of transforming growth factor β1 and β2. Biochim. Biophys. Acta.

[29] Tirino V., Camerlingo R., Bifulco K., Irollo E., Montella R., Paino F., Sessa G., Carriero M.V., Normanno N., Rocco G. (2013). TGF-β1 exposure induces epithelial to mesenchymal transition both in CSCs and non-CSCs of the A549 cell line, leading to an increase of migration ability in the CD133^+^ A549 cell fraction. Cell Death Dis..

[30] Remuzgo-Martínez S., Pilares-Ortega L., Alvarez-Rodríguez L., Aranzamendi-Zaldunbide M., Padilla D., Icardo J.M., Ramos-Vivas J. (2013). Induction of proinflammatory cytokines in human lung epithelial cells during Rhodococcus equi infection. J. Med. Microbiol..

[31] Gong L., Mim H.J., Zhu H., Zhou X., Yang H. (2012). P-selectin-mediated platelet activation promotes adhesion of non-small cell lung carcinoma cells on vascular endothelial cells under flow. Mol. Med. Rep..

[32] Li X., Tai H.H. (2013). Thromboxane A2 receptor-mediated release of matrix metalloproteinase-1 (MMP-1) induces expression of monocyte chemoattractant protein-1 (MCP-1) by activation of protease-activated receptor 2 (PAR2) in A549 human lung adenocarcinoma cells. Mol. Carcinog..

[33] Lee I.T., Lin C.C., Lee C.Y., Hsieh P.W., Yang C.M. (2013). Protective effects of (−)-epigallocatechin-3-gallate against TNF-α-induced lung inflammation via ROS-dependent ICAM-1 inhibition. J. Nutr. Biochem..

[34] Feng L., Jia X.B., Jiang J., Zhu M.M., Chen Y., Tan X.B., Shi F. (2010). Combination of active components enhances the efficacy of Prunella in prevention and treatment of lung cancer. Molecules.

[35] Yu J.H., Zheng G.B., Liu C.Y., Zhang L.Y., Gao H.M., Zhang Y.H., Dai C.Y., Huang L., Meng X.Y., Zhang W.Y. (2013). Dracorhodin perchlorate induced human breast cancer MCF-7 apoptosis through mitochondrial pathways. Int. J. Med. Sci..

[36] Yan Y.Y., Bai J.P., Xie Y., Yu J.Z., Ma C.G. (2013). The triterpenoid pristimerin induces U87 glioma cell apoptosis through reactive oxygen species-mediated mitochondrial dysfunction. Oncol. Lett..

[37] Yanagisawa S., Koarai A., Sugiura H., Ichikawa T., Kanda M., Tanaka R., Akamatsu K., Hirano T., Matsunaga K., Minakata Y. (2009). Oxidative stress augments toll-like receptor 8 mediated neutrophilic responses in healthy subjects. Respir. Res..

